# Ultrafine particle levels measured on board short-haul commercial passenger jet aircraft

**DOI:** 10.1186/s12940-021-00770-7

**Published:** 2021-08-17

**Authors:** Susan Michaelis, Tristan Loraine, C. V. Howard

**Affiliations:** 1 Occupational and Environmental Health Research Group, University of Stirling, Stirling, UK; 2 Global Cabin Air Quality Executive, London, UK; 3 Centre for Molecular Biosciences, University of Ulster, Ulster, UK

**Keywords:** Ultrafine particles, Bleed air, Oil contamination, Oil fumes, Aircraft air supply

## Abstract

**Background:**

Airline crew members report adverse health effects during and after inhalation exposure to engine oil fumes sourced to the air supply system onboard commercial and military aircraft. Most investigations into the causal factors of their reported symptoms focus on specific chemical contaminants in the fumes. The adverse health effects reported in aircrew exposed to the aircraft air supply, bled unfiltered off the engine or Auxiliary Power Unit (APU) may be related to particulate exposures, which are widely known to effect health. While oil contaminates the aircraft air supply, some suggest that this will only occur when there is a bearing seal failure, others document that there is low level oil contamination of the air supply during normal engine operation. This brief pilot study explores whether particulate exposure may be associated with the normal engine/APU and air supply operation and to therefore increase the understanding that UFP exposures may have on crew and passengers.

**Methods:**

An ultrafine particle counter was utilised by an experienced airline captain in the passenger cabin of four short-haul commercial passenger aircraft. All flights were under 90 min on aircraft from two different carriers ranging from 7 months to 14 years old.

**Results:**

UFP concentrations showed maximum concentrations ranging from 31,300 to 97,800 particles/cm^3^ when APU was selected on as a source of air on the ground and with engine bleed air and the air conditioning packs selected on during the climb. In 2 of the 4 flights the peaks were associated with an engine oil smell. Increases in UFP particle concentrations occurred with changes in engine/APU power and air supply configuration changes.

**Conclusions:**

This study identified increases in UFP concentrations associated with engine and APU power changes and changes in air supply configuration. These results correlated with times when engine and APU oil seals are known to be less effective, enabling oil leakage to occur. The concentrations reached in the passenger cabins exceeded those taken in other ground-based environments. UFP exposures in aircraft cabins during normal flight indicates there will be health consequences for long serving aircrew and some passengers.

## Background

It is increasingly recognised that chronic exposure to particulates are associated with a range of adverse health conditions [1, 2]. Health outcomes associated with exposure to UFP’s are suggested to be more toxic than larger particles [3].

Adverse effects being reported by aircrew in relation to exposures to aircraft supply air contamination include a range of both acute and chronic effects that is being labelled primarily as a discrete occupational health condition increasingly termed ‘Aerotoxic Syndrome’ [4–9]. The diffuse pattern of acute and chronic effects include the following areas: neurological, neurobehavioural, respiratory, cardiovascular, gastrointestinal, general (fatigue, rheumatological, chemical sensitivity, performance decrement, soft tissue), irritant and skin [6]. An increased vulnerability for aircrew associated with continual low dose exposures in addition to less frequent acute on chronic higher dose events may explain the differential susceptibility between aircrew and passengers being reported [6, 7].

While there are no sensors in aircraft to detect contamination of the breathing air supply, there have been a variety of ad-hoc studies undertaken over recent years. These have primarily looked at contamination by individual compounds and analysed these in terms of occupation exposure limits (OEL). However more recently there has been increasing focus on exposure to particulates in the low PM range and UFPs.

On all current commercial jet aircraft apart from the Boeing 787 Dreamliner (B787), the air supplied for passengers and crews to breathe and to pressurise the aircraft in-flight, is provided from the engines, unfiltered, in a process known as ‘bleed air’. The process became common place in the late 1950s, early 1960s, on all commercial jet aircraft. Crew health and flight safety concerns associated with exposure to bleed air contaminants date back to the early days of bleed air and the introduction of synthetic jet engine oils (which replaced mineral oils). These concerns have become more widely discussed over time. Concerns have also been raised in relation to air supply contamination by hydraulic and de-icing fluids.

In recent years, there have been a number of air monitoring studies and swab testing analyses [10–13]. These have identified the presence of the organophosphates typically used in jet engine oils and hydraulic fluids, such as tricresyl phosphate (TCP) and tributyl phosphate (TBP) in cabin and flight deck air. Additionally, the complex mixture of pyrolysis products released when the oils and fluids are heated has also been frequently identified [12, 14–16]. Many within the aviation industry state that contaminated air events only occur in rare failure conditions and that the measured concentrations are too low to pose any harm, or any contaminants found come from elsewhere, such as plastics, electronics or interior furnishings.

More recently there have been several studies, which have simulated oil leakage or measured particulate size and or concentration in the aircraft cabin air with a focus on UFPs [10, 11, 17–21].

Recent research has identified that low-level oil contamination of the air supply is a factor of current engine design [22–24]. Leakage of oil from the engine or APU oil-bearing chambers is an expected occurrence in current engine designs using compressor sourced pressurised air for chamber sealing. In addition to chronic low-level contamination, higher levels are expected to occur during transient engine power and/or air supply configuration changes and less frequently during system malfunction or seal failure. When background levels increase to a level detectable by airline crews or passengers, these become known as ‘fume’ or ‘contaminated air’ events. These exposures may be the cause of the reported but not universally accepted condition that is sometimes referred to as ‘Aerotoxic Syndrome’ [6, 7, 25].

We therefore decided to undertake a small proof of concept study to further investigate if there may be any correlation in UFP levels and phase of flight or engine/APU power or air supply settings, and to compare UFPs levels on aircraft to other environments. An additional research question was to see if UFPs could potentially be a good marker of oil contamination of the bleed air in the future.

## Methodology

The primary aim of this study was to utilise an ultrafine particle counter in short-haul passenger aircraft to assess whether there is a correlation between UFP count and engine/APU power settings and air supply changes. The secondary aim was to determine if UFPs may be a useful real-time marker of oil contamination in the aircraft bleed air supply.

A TSI P-Trak® Ultrafine Particle Counter (UPC) 8525 was used for carrying out all UFP measurements. Initially, the device was taken to four different ground-based locations. Then, measurements were collected during four short scheduled domestic passenger flights by an experienced airline captain. All four flights were on Airbus A320/A319 aircraft with all sectors under ninety minutes. Two different carriers were selected, with aircraft age ranging from seven months to fourteen years. The device detects particles in the 20–1000 nm range and was set up to data log every second. The device is ready to use after a one-minute auto calibration phase and records particles per cm^3^. The device was situated in the passenger cabin under a passenger seat beside a window at all times and on all flights. It was activated once the researcher was seated and data logged every second until the aircraft returned to the gate after landing and the passenger doors were opened.

The captain who collected the samples noted the tail/registration number of each of the four sampled aircraft and, subsequently, located the date of the first flight and thus, the age of each aircraft via public, online sources. However, the number of operating hours for the engines and the APUs installed on those four aircraft are not publicly available and the age of each engine/APU may not equate to the aircraft age.

## Results

Measurements in other non-aviation environments are shown in Table 1 and ranged from 2,428 particles per cm^3^ to levels ten times higher outside Victoria station in Central London at 24,428 particles per cm^3^.

**Table 1:** UFP Particle count in various non aircraft locations

Location	Average level recorded particle/cm^3^
Beach beside water – English Channel	2,428
Train compartment – Moving train	3,242
Household kitchen	3,661
Street outside Victoria station—London	24,428

Table 2 outlines the aircraft model, first recorded flight and engine type. The samples were all taken on either Airbus A319 or A 320 aircraft with the aircraft age ranging from 7 months to 14 years old.

**Table 2:** Aircraft and engine details for flights undertaken

Flight	Airbus model	Aircraft’s year of first flight	Engines
1	A320neo	2017	2 × CFMI LEAP
2	A320	2014	2 × CFMI CFM56
3	A319	2006	2 × IAE
4	A319	2004	2 × IAE

Maximum and minimum levels measured on board commercial jet airliners ranged from 35 to 97,800 per cm^3^ as shown in Table 3 below.

**Table 3:** Particle counts/cm^3^ at various phases of aircraft operation

Flight	Max / Min Reading particle per cm^3^	Phase of flight of maximum peak reading
1	96,700 35	Peak occurred with associated noticeable oil smell on ground after flight when APU air supply was selected on. Levels increased from 9,510 to 96,700 in 33 secs
2	31,300 76	Peak immediately after engine bleed air/packs selected on after climb power selected. APU was not used on the ground
3	81,800 147	Peak occurred with associated strong oil smell on ground after flight when APU air supply was selected on. Levels increased rapidly to 81,800
4	97,800 893	Peak immediately after engine bleed air/packs selected on after climb power selected Device stopped operating mid-flight due to power failure

**Fig. 1:**
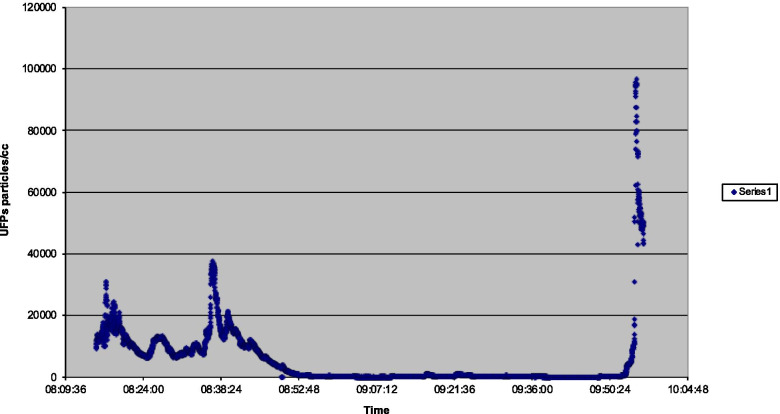
Flight 1

Flight 1 (Fig. 1) showed that a peak UFP level of 96,700 particles per cm^3^ occurred with an associated noticeable oil smell on the ground after flight, when the APU air supply was selected to supply aircraft cabin air on the ground with the doors still closed. Two lower peaks of 31,300 and 37,600 were in relation to the introduction of APU bleed air on the ground before engine start and after the introduction of engine bleed air after a reduced power takeoff respectively. The first peak of 31,300 saw an increase from 10,000 in 16 s. The second peak of 37,600 saw an increase from 14,400 in 43 s.

**Fig. 2:**
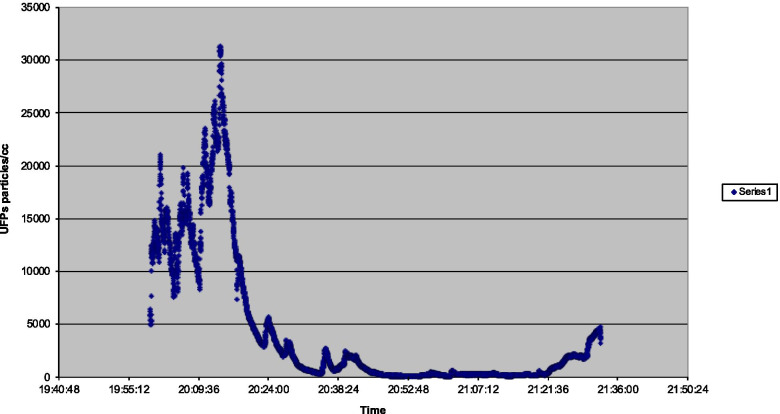
Flight 2

The peak UFP measurement in flight 2 (Fig. 2) occurred at 31,300 after the introduction of engine bleed air after a reduced power takeoff. A secondary peak of 21,100 (increase from 11,600 in 15 s) was associated with the introduction of APU bleed air supply prior to engine start. No APU was used after landing.

**Fig. 3:**
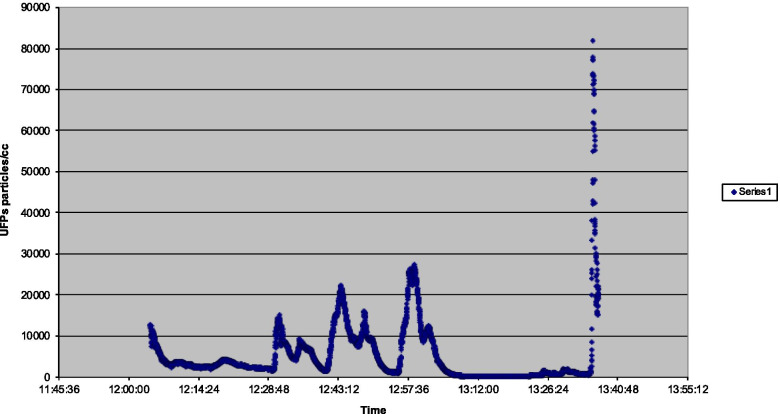
Flight 3

The maximum UFP peak in flight 3 (Fig. 3) of 81,800 was linked to a noticeable oil smell noticed on the ground, after flight, when APU air was selected on. Levels increased from 1,100 to 81,800 in 35 s. Additional lower peaks of 14,300 after the introduction of engine bleed air after a reduced power takeoff, and peaks of 22,300 and 26,300 when climb power was re-selected during the climb phase of flight after temporary reductions in engine power associated with a reduction in aircraft rate of climb.

**Fig. 4:**
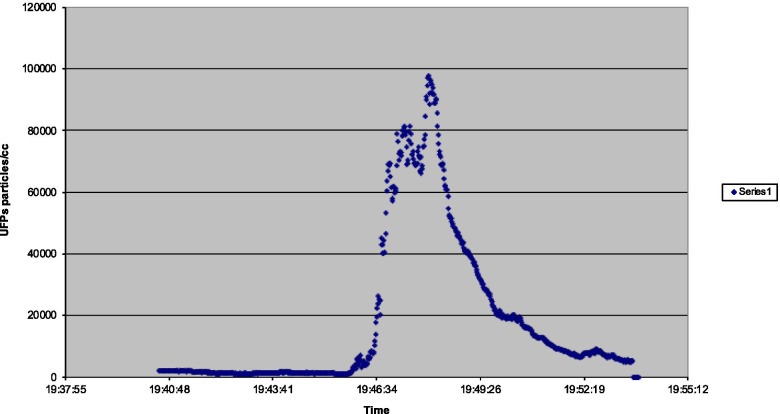
Flight 4

The peak in flight 4 (Fig. 4) occurred at 97,800 after the introduction of both air conditioning packs (supplied with engine bleed air) after a full power takeoff. An additional short secondary peak of 81,500 occurred prior to this when the first of the two air conditioning packs was selected on after takeoff once climb power had been selected. This was an increase from 7,740 to 81,500 in 50 s. Data collection stopped during the climb due to a power failure.

Table 4 summarises the UFP concentration levels measured in this study, their phase of flight, whether a smell was noticed and aircraft details as available.

**Table 4:** Study results summary

Flight	Maximum UFP count (000)/cm^3^/ phase of flight	Secondary peak and phase of flight	Age of aircraft	Potential maximum age of engine	Oil smell detected
Flight 1	96.7 – APU selected on after landing	37.6 After the introduction of engine bleed air after a reduced power takeoff	6 months	2 years	Yes – APU selected on after landing
Flight 2	31.3 – After the introduction of engine bleed air after a reduced power takeoff	21.1 Introduction of APU bleed air prior to engine start	3.5 years	22 years	No
Flight 3	81.8—APU selected on after landing	26.3 Climb power reapplied after level off during climb	12 years	21 years	Yes – APU selected on after landing
Flight 4	97.8 – After the introduction of engine bleed air after a full power takeoff	81.5 After 1^st^ pack selected on after takeoff	14 years	21 years	No

## Discussion

### Background

There is increasing data available outlining substances in the cabin and bleed air in normal operations [26]. A thorough analysis was undertaken by the manufacturer of an engine used on a flight into Malmo Sweden, in which two Swedish pilots were temporarily totally incapacitated whilst flying a British Aerospace BAe 146 aircraft in the descent to Malmo, Sweden. The investigation found that an oil leak occurred in which a complex range of substances were identified in the bleed air used to supply the breathing air in the cabin. [27, 28] Despite this data being available, the debate continues as to which individual substances could be measured in real-time to confirm the presence of heated oils or hydraulic fluids in the air supply that may be linked to reported ill health effects [29, 30].

Despite the ‘Malmo’ data being available to the European Commission and European Aviation Safety Agency (EASA), in 2017 they undertook a large-scale study to determine which contaminants were associated with ‘fume’ events in order to develop mitigation strategies [31]. However, this work did not achieve its research objectives with no final report published to date [31, 32]. In the United States, the Federal Aviation Administration (FAA) failed to deliver on a law that required the agency to lead research and development work into bleed air sensors. They instead claimed that fume event occurrences are extremely low and *"the specific nature and extent of potential decomposition reactions of engine oils and hydraulic fluids are largely unknown”* with the resultant potential toxicity *“highly speculative”* at present [33].

Van Netten et al. [14] established that that some of the chemicals in oil fumes will vary according to the temperature to which the oil is heated, thus creating a complex pyrolysed mixture. Others suggest that, *“little is known about the characteristics of the aerosol resulting from oil contamination of bleed air”* [18]. This has therefore become a growing area of interest.

Jones et al. (2017) identified that pyrolised jet engine oils release UFPs (diameter less than 100 nm) in most normal engine operations [17]. It was therefore suggested, *“development of sensors for detecting oil contamination in aircraft bleed air should focus on ultrafine particle detection”* [17].

In 2017 Michaelis et al. [6] and Howard et al. [7] identified a pattern of effects related to acute and chronic exposures to bleed air contamination. Howard et al. (2018) suggested that a large part of the causal mechanism for ‘Aerotoxic Syndrome’ may be the exposure to organophosphates and a wide range of Volatile Organic Compounds (VOCs) that bind with nanoparticles which then act as Trojan horses to transport the chemicals across the blood brain barrier which then enter the brain [25].

A 2019 paper by Fushimi et al. studied size-resolved particulate samples in ambient air near a runway at Narita Airport in Japan [34]. They reported *“we clearly demonstrate that organic compounds in the ambient nanoparticles (diameters:* < *30 nm) were dominated by nearly intact forms of jet engine lubrication oil”* [34]. The vented lubrication oil was reported to be mixed with hot combustion gases in the exhaust area or in the atmosphere. This is of considerable importance because the process that generates particles in the high temperature oil spaces of the engine, escaping via the breather or oil seals into the engine exhaust, will be exactly the same for the particles appearing in engine bleed air.

Despite the issue being known within the aerospace industry for over 65 years, neither military nor commercial jet aircraft have any form of bleed air contamination air monitoring systems fitted, to warn when the air supply is contaminated.

### Air monitoring studies: VOCs

Most previous air sampling studies have focused on VOCs and have not considered particles.

Earlier studies addressing VOCs have analysed the results in terms of Occupational Exposure Limits (OEL). This has continued to date despite OELs not being available for many substances, not set for synthetic jet engine oils and not applicable to passengers or the aircraft environment [35–37].

As an example, in the 1999 Malmo event referenced above, [27] after landing, one of the engines was identified as having an oil leak and the engine was sent to the manufacturer for analysis. The engine manufacturer measured the substances in the engine bleed air and provided this data to the Swedish Accident Investigation Authority. The bleed air data [28] identified a complex mixture of chemicals at low levels and below individual OEL/threshold levels (where they exist) consistent with heated engine oil.

Previous VOCs measurement studies in aircraft cabin generally have not identified the actual source of contamination and usually have not provided any correlation to engine power settings or the air conditioning system configuration status. This has led to an ongoing debate about which markers are best suited to identify sources of air supply contamination and which substances may cause harm. Recently there has been an increased interest in the potential of UFPs as a rapid means to identify bleed air contamination.

### Previous air monitoring studies: particulate aerosols

Limited studies have previously shown elevated levels of lower range PM and UFPs on aircraft without investigating in depth any potential correlation between UFP levels, phases of flight and engine power settings [10, 19, 20].

In-flight measurements of particle aerosol concentrations in aircraft or ground based simulations have taken place over the last two decades, but these have primarily focused on particulate matter PM10 and to a lesser extent PM2.5 and have focussed on a mass basis rather than on number-weighted concentration. Limited but more recent work has focused on lower levels of PM and UFPs.

For example, Jones et al. undertook a four-part experimental program to develop a detailed characterisation of particles that result when bleed air is contaminated with lubricating oil [17]. The researchers noted: *“The measurements showed that oil contamination in the compressor will result in a fog of very fine droplets in the bleed air under most operating conditions. Typically, these droplets are in the 10–150 nm range. With very low contamination rates, it appears that many of the droplets may be even smaller than 10 nm”* [17]. Peak concentrations of particles were identified in the range of 50-70 nm. It was concluded that based on the experiments, *“oil contamination leads to a large number of particles in the bleed air”* [17]. Previous similar studies with the same group identified a “*substantial increase of ultrafine particles as the temperature is increased to the maximum temperatures expected during normal aircraft operation”* [18].

Li et al. suggest that airborne particles are an important type of air pollutant in aircraft cabins [19]. Measurements larger than 0.3 µm were made in the supply and breathing air on nine short haul flights [19]. The study identified that particles (> 0.3—2 µm) in the breathing zone came mainly from unfiltered bleed air. The highest particle concentrations were recorded in the 0.3—0.5 µm size range. It was reported that particle concentrations in the supply air related to atmospheric environment of the flight route and that particles increase in the supply air when entering cloud and during turbulence. Increased particle size (> 2 µm) was attributed to both the bleed air and cabin interior. Particle concentration (PM > 0.3) increased in the supply air and breathing zone during pre-taxi, taxi, take off, descent phases as well as in cloud and during turbulence.

Crump et al. undertook air quality studies, including UFP particle concentration measurements, on 100 flights on five different aircraft types [10]. A TSI P-Trak® Ultrafine Particle Counter (UPC) 8525 was utilised, identifying particles from 20–1000 nm/m^3^ (0.02-1 µm/m^3^)[10]. However, the study focussed on UFP measurements rather than particle matter (PM) as the reports stated “*particle number is generally considered to be dominated by the ultrafine fraction”* [10]. The data from the Crump et al. reported high levels of UFPs mainly associated with phases of flight (including ground operations) other than the cruise, but made no correlation to engine power settings or air conditioning system configuration. The study identified that UFP concentrations showed increases during initial ground operations prior to flight, start up, take off and climb, and again at the end of flight and taxiing in. During five of the 100 flights, the maximum count of the instrument (500,000 particles cm^3^) was exceeded. 65% of the flights recorded maximum particle concentrations per cm^3^ of 100,001—> 500,000, 22% were 50,001—100,000 particles cm^3^ and 13% were > 10,001—50,000 particles cm^3^. Aircrew and researchers were requested to report any air quality events during the flights. Fumes or smells during the flight were detected by the crew or researchers in 38% of the flights with the predominant descriptor being ‘oil’ or ‘oily’ in 26% of the 100 flights. However, these were said to be “*below the threshold where reporting would be required”* [10]. Therefore, these are likely to have been lesser type events, without maintenance defects identified or major impairment, despite all cases of fumes or contaminated air events requiring to be reported under the European regulations [38]. 25 of the 100 flights measuring UFPs encountered an air quality event according to the researchers, of which 19 showed maximum counts of 100,001—> 500,000 particles cm^3^ and five recorded maximum counts of 50,001—100,000 particles cm^3^.

In 2012, Spengler et al. measured UFPs, identifying a 15-min maximum of up to 312,000 particles/cm^3^ [20]. Together, the presence of ambient tropospheric ozone and meal service in the passenger cabin were suggested as the primary causes of longer duration elevated levels of UFP. However, the authors also noted that *“episodic variation in UFP might be associated with switchover in bleed air from pressure in the Environmental Control System or variations in engine power. However, it was not possible to discern these events during the course of air monitoring in the cabin”* [20].

A 2017 EASA cabin air quality study measured UFPs during 69 commercial flights on five different aircraft types [11]. Phase of flight detail was not provided for all data, but the authors noted that elevated counts of UFP were primarily associated with descent/approach, taxiing, the start and end of flight and time on the ground. Measurements collected on the B787 Dreamliner (which does not use engine bleed air) were detectable, during the first 2 h of measurements, suggesting that the primary source was external air pollution. A long-haul aircraft may sit on the ground with the air supply on for an extended period before take-off.

A recent aircraft UFP measurement study found that an increase of UFP levels occurred within the cabin due to external particles (ingress of ambient pollution outside the aircraft) or airframe events (sourced to the aircraft or engines/APU) [21]. The latter were often associated with changes in engine conditions. Over 50% of these were observed on approach and landing with no increase of outboard concentrations, with it *“speculated that these events could be from the bleed air systems, as seal integrity may change under these conditions”*. Aerosol composition data showed non exhaust, non airport sources influence the cabin air quality, including the presence of lubrication oil [21].

Using the same measuring equipment as the Crump 2011 study, our research study undertaken in routine flights, with no crew reported fume events, was able to show a clear correlation between UFP levels and engine power settings and air conditioning system configuration.

The UFP peaks in three of the four flights we undertook were above 80,000 particles/cm^3^ and the maximum above 97,000 particles/cm^3^. The primary and secondary peaks were all associated with engine power changes or the introduction of air conditioning air from the APU or engines. (The term ‘engine’ may be used below to address both the main turbine engines as well as APUs.) The peak UFPs identified were seen most commonly during the following phases of flight: introduction of APU bleed air, switching on of air conditioning packs after takeoff (after climb power selected), taxiing out, re-application of climb power during climb after a temporary level off and in the descent with engines at idle. The least level of UFPs were identified during steady state engine operation, which is during the cruise phase of flight. Our data shows frequent rapid decreases in UFP levels, which is to be expected with the high air exchange rate in aircraft.

Ultrafine particles are of course sourced to various environments, however the peaks identified in this brief study are most likely associated with pyrolysed oil by-products entering the aircraft cabin from the bleed air supply either from the engines or from previously contaminated bleed air ducting.

### Oil seal leakage

Low levels of oil leakage past seals in normal operations are increasingly recognised to occur. Michaelis (2016) identified that jet engine seals reliant upon compressor generated pressurised air for sealing the engine oil bearing chambers allows low levels of oil to leak past the seals, back into the compressor airstream as a function of design [22, 23]. These findings were supported by Howard et al. (2018) [25]. Oil leakage occurs at very low levels during normal engine operation as dynamic oil seals are not an absolute design and are designed to leak, or limit leakage, rather than prevent it [23, 25, 39]. While seals are used to limit oil escape, they are not completely leakproof and other design factors are also used to limit leakage [40]. Chupp et al. (2006) reports that *“a zero leakage seal is an oxymoron”* [41].

Whilst seal effectiveness is maximised for steady state operation (the cruise phase of flight) increased leakage rates may occur with transient changes in speed, temperatures and pressures of the engine [22–25]. Michaelis’s findings of 2016 [22, 23] are entirely consistent with the data from our study. This leakage is within what the engine manufacturers determine as within a normal *‘permissible oil consumption’* limits. Exxon Mobil and Rolls Royce have recognised the loss of oil past engine seals during normal operation or as part of the permissible loss of oil [42, 43].

In 2019 Johnson reports that *“most turbine engines lose some lubricant under normal operating conditions’*” and *“some of the leaked material can be transmitted into the passenger cabin as both vapors and nano-droplets”* at low levels during normal flight [44]. An EASA cabin air quality study reports that *“most engines might have a certain turbine oil leak rate”*, also described as *“permanent engine oil (contaminant) entry into the cabin”* [11]. Additionally, the study reported that: oil fume events were reported during changes of engine thrust settings and that *“detection of concentration peaks of submicron aerosols in the bleed air could therefore be a hint on oil leaks in the engine”* [11]. An Airbus report highlights that *“in the case of oil contamination from the engine, it is also quite possible that oil smells would become more apparent under certain pack configurations’*” and that is *“expected that reports of odour would be associated with changes in engine speed or bleed system configuration”* [45].

There is also further recognition that oil seals are less effective during transient engine operation [20–22, 41, 46–51] and that there is a constant chronic exposure to oil leaking through the seals [7, 11, 39, 51]. Howard et al. (2017) also identified that oil fumes, as a complex mixture, enter the aircraft air supply as either acute fume events or chronic low-level exposures on a day-to-day basis [7].

### Oil leakage and UFPs

It is also increasingly accepted that pyrolysed engine oils will generate UFPs in normal engine operation [17, 25]. Howard et al. stated that “*the physical and chemical nature of engine oils and the high temperatures attained in aircraft jet engines (up to 1,700 °C in the oil circulation and up to 30,000 °C in the bearings) explain why UFPs are to be expected”* [25]. This was supported by research by Jones et al. that simulated oil injection into engine compressors showing *“a fog of very fine droplets in the bleed air under most operating conditions’*” was generated [17]. Williams et al. (2021) [21] reported in board generated UFP events occurred most commonly when the engines were changing conditions, with speculation that these were associated with bleed air systems and oil leakage when seal integrity may change, particularly during the approach and landing [21].

Our study clearly identified increased UFP concentrations during engine power and air supply configuration changes. This correlates with when oil sealing is less effective. Li et al. identified smaller PMs associated with the bleed air supply at the same phases of flight as our study as well as during turbulence and in cloud [19]. Li’s reference to cloud may be in part linked to the hydrolysis of the engine oil in contact with moisture that is reported to generate a reported dirty sock smell. Spengler et al. also suggested higher UFP concentrations may be associated with changes in air supply and engine power [20].

The smaller PMs and UFPs are recognised as associated with bleed air supply and leakage of the turbine oils as distinct from other sources of UFPs and PMs [17, 19]. Jones et al. found that droplets of oil are in the 10–150 nm range and possibly smaller, while the peak concentrations of particles were identified in the range of 50-70 nm [17]. Li found increased particle counts with smaller particle size, and that particles in the supply air mainly came from the bleed air [19]. It is now suggested that sensors for oil contamination supplied via or generated within engines should focus on UFP detection, capable of detecting very small UFPs, 10 nm and below [17, 52].

An increasing yet not fully appreciated understanding involves the oil aerosols being deposited within the air ducting and then possibly revolatising and entering the air supply with changes in thermo-mechanical conditions [11, 22, 53]. EASA suggests the release of oil sourced contaminants from the ducting may occur with *“mechanical or thermal stress or the introduction of solvents such as water or de-icing agents”* [11]. An FAA report recognised the re-release of pyrolysed oil products from the air supply ducting during *“times of high demand of cabin heat and/or by physical disturbances and/or stresses occurring during flights, particularly during taking off and landing”* [53]. Changes in airflow and moisture during periods of rain, anti-ice and turbulence can also affect leakage rates [22]. Additionally, when jet oil esters (base stock of the oils) are exposed to water, including water from the atmosphere hydrolysis occurs, forming acids, thus degrading the oils, creating odours over time [54, 55]. Therefore, the smaller PMs and UFPs identified by Li et al. may not be solely related to cloud particles and could also be a factor of oil exposed to water.

Although seal leakage is expected to increase with sealing wear linked to engine age, our study was not able to provide a clear correlation between engine age and increased concentrations of UFPs. The study was however able to show that aircraft with new engines still show raised levels of UFPS in the aircraft cabin in varying stages of flight as shown in Table 4.

At present there are no commercially available sensors readily available with demonstrated ability to reliably detect oil contamination of bleed air [17]. UFP measurements as a surrogate for oil contamination in the bleed air supply offers a promising way forward over conventional measurement of individual chemical compounds.

### Contaminated bleed air and adverse health effects

There is increasing recognition that a range of acute and chronic adverse effects are associated with reports of aircraft bleed air contamination and fume events. The first reports date back to the 1950s soon after synthetic engine oils were introduced to turbine engines [56]. Michaelis et al. reported that in a review of 15 aircraft fume events, 80% of the events related to non-visible fumes only; all occurred in transient engine/APU operation; 80% occurred during the climb or descent phase and 87% were linked to positive maintenance findings of oil leakage into the air supply [6]. Adverse symptoms ranging from impairment to incapacitation were reported in 93% of the events with 73% involving the pilots. 75% of the events included a wide range of adverse effects in 1 or more crew members, while 53% of the events involved long-term adverse effects in 1 or more crew members. Nine of the pilots either became unfit to fly or died. Passengers reported adverse effects far less frequently than the aircrew, in 27% of the events [6]. The pattern of symptoms reported included a diffuse range of acute and chronic effects include the following areas: neurological, neurobehavioural, respiratory, cardiovascular, gastrointestinal, general (fatigue, rheumatological, chemical sensitivity, performance decrement, soft tissue) irritant and skin [6]. Similar patterns have been increasingly reported elsewhere [4, 5, 9, 57–68]. The FAA reported a range of similar adverse effects as well as delayed effects could be expected following exposure to the complex mixtures associated with pyrolysed jet oils [53]. This report noted that while some individual components may not be toxic at the levels identified, they could become highly toxic when combined. Howard et al. suggested that when the neurological signs and symptoms reported by Michaelis et al. were considered together, they constituted a group of non-localising functional deficits consistent with a diffuse encephalopathy [6, 25]. The pattern was reported to be similar to sheep dippers flu with the common feature being organophosphates [25].

Michaelis et al. found a clear cause and effect relationship when taking into account symptoms, diagnoses and findings in relation to the occupational environment [6]. This was described as a new occupational disease in a similar manner to the 2005 reporting of the *“development of an irreversible discrete occupational health condition”* [4, 6]. A clear cause and effect relationship between exposures and acute and chronic adverse effects and inadequate medical management has been reported elsewhere [7, 67, 69].

The continual low dose exposures to fugitive emissions in normal operations combined with acute fume events released via the cabin air supply system may explain the differential susceptibility being identified between aircrew and passengers [7]. The consistency of the findings between flights in our study, and the correlation of our data with other UFP studies leads to the reasonable conclusion that what is being observed represents 'normal' operating conditions on the current fleet of civil aircraft. From our current understanding of the effects of chronic exposure to UFPs this implies that there will inevitably be health consequences for long serving air crew. However, passengers occupy the same environment as aircrew and frequent flyers may also spend considerable time in aircraft. In 2018, it is reported that 4.3 billion passengers travelled on scheduled services, operated by in excess of 0.5 million aircrew [70].

The aviation industry has continued to question which specific chemicals may be responsible for ill health with regard to the breathing air supplied in aircraft [31, 32, 71]. This has been based upon a number of ad-hoc cabin air monitoring studies that have not captured significant fume events and which have then been interpreted in light of occupational and other exposure limits [10, 11, 13]. Outcomes have suggested all levels identified are better than in other environments such as homes and offices and are all within exposure limit guidelines. However, this fails to take into account that most measurements are not collected during fume events. Also comparing these measured concentrations to OELs fails to take into account that there are not OELs available for some of the key substances of concern and that they should not be used in the aircraft environment or with complex thermally degraded mixtures [36]. Additionally there has been an inappropriate focus on Tri-ortho-cresyl phosphate (ToCP) and Organophosphate Induced Delayed Neurotoxicity [72]. As seen, there has more recently been a move towards the measurement of UFPs in aircraft supply air.

There is wide acceptance that chronic exposure to particulate aerosols damages health [1, 2]. It has been estimated that poor air quality is responsible for at least 40,000 deaths per year in the UK [73]. These studies measured outcome against PM 2.5 for which there is no ‘no effect’ level, as any exposure is known to cause harm [1].

The use of UFP measurements is only now starting to gather some interest. Prior to this time, particles have been studied in terms of mass, rather than size and concentration. From our knowledge of the physico-chemical properties of nanoparticles, their surface chemistry and enhanced reactivity it has long been argued that they would be more toxic than larger particles. The work of Wichmann and Peters supports this [3]. They showed that UFP concentration was more predictive of harm through cardiovascular accidents than PM 2.5 concentration in a study on the population of Erfurt in Germany [3].

Howard et al. discussed the likelihood that UFP concentrations on cabin air could be implicated in the aetiology of Aerotoxic Syndrome [25]. This was mainly predicated on the formation of OP compounds on the surface of carbon-based particles associated with high engine temperatures. The continual presence of UFPs over the extended periods aircrew spend in the aircraft environment, will predispose them to chronic respiratory problems and exacerbate the translocation of neurotoxic substances across the blood brain barrier [25].

The findings of Fushimi et al. that the UFP fraction of particulate aerosols produced by aircraft gas turbine engines predominantly consists of oil droplets is of enormous significance [34]. This would boost the dose of triaryl phosphates (TAPs) from inhaled droplets by orders of magnitude. It would become the major source of exposure. This pilot study demonstrated that aircrew and passengers are exposed regularly to concentrations of particles well in excess of those found in major urban traffic sites. In addition to the underestimation of the toxicity of the ortho isomers of TCP, other than ToCP, by a factor of around 6 million, the OPs adherent to particles in the aerosols in aircraft cabins, which are not measured, is of great importance [72].

All studies of exposure to toxic substances in aircraft cabin air to date have focused on the vapour phase content of the organophosphates present [72]. This paper and recent other research suggest that investigators may have been looking in the wrong place. It is clear that chemical analysis of the particulate aerosols in engine bleed air is urgently required.

### Study limitations

There are several limitations with the present study that should be addressed. The P-Trak 8525 UFP counter measures particle sizes including UFPs up to PM1 (20–1000 nm) and therefore is not exclusive to UFPs. However, it does not differentiate between the sizes. As discussed previously the particle concentrations were highest at UFP sizes or 50 to 70 nm [17], increased particle concentrations were associated with the smaller PM < 0.3 mainly from the bleed air supply [19] and UFPs were deemed to be dominant over PMs [10]. Future studies should utilise equipment that measures UFPs only and/or one that can differentiate the particle sizes.

It was deemed not possible to gain airline consent and participation in this small pilot study and therefore detailed information as to the engine power or air supply settings were not available. However, the measurements were undertaken by an experienced commercial airline Captain and therefore a good understanding of the phase of engine and air supply phases of operation was available. It would be beneficial for future studies to gain airline support, so that the specific engine/APU operations and phases of flight, along with air supply configuration, ambient conditions on the ground and other cabin or environmental factors could be recorded.

Other unknown factors include the actual age of the engines and maintenance schedule for the engine/APU and air supply systems or if the aircraft was operating with any known maintenance approved defects. Table 2 defines the age of the aircraft, but it can only provide the introduction of the engine type into airline operation, rather than actual age of the specific engine. Additionally, no data for the age/condition of the APU was available. Another interesting point is the greatly reduced levels of particles identified in the short cruise phase of the flights. Generally, this is expected as the engines are at a steady state operation which optimises sealing efficiency conditions. The concentrations during the cruise in our limited study were generally several hundred particles/cm3 or less, a similar finding as in the Cranfield [10] study which used the same equipment and also not greatly dissimilar to the Li study [19].

The validity of the low levels recorded in the cruise flight sectors was raised with the manufacturer. It was advised that when the aircraft cabin altitude in flight reaches its maximum of 8,000 feet, the air pressure drops to about 750 mbar, which will impact (shift) the P-Trak’s counting efficiency curve and the P-Trak would most likely not be accurate under those conditions but at least the data will show trends and event.

Additionally, no data was available regarding relative humidity, but the instrument manufacturer advised that, the Kelvin diameter that is responsible for the activation of small particles in a CPC instrument like the P-Trak is theoretically independent of RH.

## Conclusion

This small proof of concept study sought to investigate if there was any correlation in UFP levels and phase of flight or engine power setting, and to compare UFPs levels on aircraft to other environments. An additional research question was to see if UFPs could potentially be a good marker of oil contamination of the bleed air in the future.

The measurement of UFPs sourced to the aircraft ventilation air supply may be a worthwhile indicator of oil contamination, whether sourced to engine or APU generated bleed air.

This study showed higher levels of UFPs in passenger aircraft compared to other ground-based environments.

The study supports the findings from the previous cabin and bleed air studies, as well as the findings that pyrolysed engine oil generates UFPs. Our study clearly identifies increases in UFP concentration during changes in engine speed and power, and changes in air supply configuration or source. This confirms that oil seals are less effective during changes of power, and concentration levels show a correlation with changes in air conditioning system configuration.

There is an emerging consistent pattern of acute and chronic adverse health effects related to exposure to aircraft contaminated air supplies in normal as well as abnormal operation. This has been labelled as Aerotoxic Syndrome. It has long been argued by some that the concentrations of triaryl phosphates (TAPS) in the vapour phase of engine bleed air are too low to be of concern in the aetiology of Aerotoxic Syndrome. The findings in this paper confirm the presence of UFP aerosols on commercial flights, often at high concentration. Fushimi et al. have reported that UFP particles emitted from aircraft jet engines *“were dominated by nearly intact forms of jet engine lubrication oil”* [34]. There will have to be a complete reappraisal of the exposure mechanisms of aircrew to TAPS in engine bleed air. UFPs are preferentially deposited in the alveolar part of the lung, where they can cross the air/blood barrier by pinocytosis. This would tend to increase TAP exposure by orders of magnitude when compared to vapour concentrations.

The consistency of the findings between flights in our study, and the correlation of our data with other UFP studies leads to the reasonable conclusion that what is being observed represents ‘normal’ operating conditions on the current fleet of civil aircraft. From our current understanding of the effects of chronic exposure to UFPs, this implies that there will be health consequences for long serving air crew and some passengers.

This research paper indicates that to enhance public health protection in aircraft using bleed air systems, it would be advantageous to filter the engine bleed and supply air to protect the diverse range of occupants that will be exposed. Future aircraft designs should no longer take the air source from the engines and APUs.

## Data Availability

The dataset supporting the conclusions of this article are included within the article.

## Abbreviations

APU: Auxiliary Power Unit
EASA: European Aviation Safety Agency
FAA: Federal Aviation Administration
Nm: Nanometer
OEL: Occupational Exposure Limits
PM: Particulate Matter
TAP: Triaryl Phosphate
TCP: Tricresyl Phosphate
ToCP: Tri-ortho-cresyl Phosphate
UFP: Ultrafine Particles
UPC: Ultrafine Particle Counter
VOC: Volatile Organic Compound
